# Evaluation of novel protease inhibitors against darunavir‐resistant variants of HIV type 1

**DOI:** 10.1002/2211-5463.12160

**Published:** 2016-11-24

**Authors:** Mari Inoue, Daiki Oyama, Koushi Hidaka, Masanori Kameoka

**Affiliations:** ^1^ Department of International HealthKobe University Graduate School of Health SciencesHyogoJapan; ^2^ Faculty of Pharmaceutical SciencesKobe Gakuin UniversityHyogoJapan

**Keywords:** antiretroviral therapy, HIV‐1, lentiviral vector, phenotypic drug susceptibility test, protease inhibitor

## Abstract

HIV disease became a manageable chronic disease since combination antiretroviral therapy (cART) was introduced as the standard treatment regimen. However, the emergence of drug‐resistant viruses is a major problem associated with cART. A phenotypic drug susceptibility test using a lentiviral vector was established and applied to evaluate new protease inhibitors (PIs). Lentiviral vectors representing a wild‐type (WT‐lentivector) and darunavir (DRV)‐resistant HIV type 1 (HIV‐1) (DRV ^r^‐lentivector) were generated. Nine clinically approved protease inhibitors (PIs) inhibited the transduction ability of WT‐lentivector similar to their inhibitory effects on the replication of WT HIV‐1. Three new PIs reduced the transduction ability of WT‐ and DRV ^r^‐lentivector, suggesting that these PIs may be the candidates as novel antiretroviral drugs against drug‐resistant variants of HIV‐1.

**Abbreviations**
APV: amprenavir
AIDS: acquired immunodeficiency syndrome
ATV: atazanavir
BOP: benzotriazol‐1‐yloxy‐tris(dimethylamino)phosphonium hexafluorophosphate
BSL: biosafety level
cART: combination antiretroviral therapy
DRV: darunavir
EDC: *N*‐ethyl‐*N*′‐[3‐(dimethylamino)propyl]carbodiimide
HIV: Human immunodeficiency virus
IC_50_: 50% inhibitory concentration
IDV: indinavir
LPV: lopinavir
NFV: nelfinavir
PI: protease inhibitor
PR: protease
RTV: ritonavir
SQV: saquinavir
TOF‐MS: time‐of‐flight mass spectrometry
TPV: tipranavir
WT: wild‐type

HIV‐1 is a major causative agent of AIDS. Combination antiretroviral therapy (cART), in use since the mid‐1990s, has improved the quality of life and life expectancy of HIV‐infected individuals, and HIV/AIDS has become a manageable chronic disease 1, 2. However, the emergence of drug‐resistant viruses has become a major problem associated with cART 3. Therefore, the development of new antiretroviral drugs is considered a critical issue. There are six classes of antiretroviral drugs: entry inhibitors, fusion inhibitors, nucleoside reverse transcriptase inhibitors, non‐nucleoside reverse transcriptase inhibitors, integrase strand transfer inhibitors, and protease (PR) inhibitors (PIs). These drugs are designed against different crucial stages of the HIV‐1 life cycle 4.

HIV‐1 PR is a viral enzyme essential for the life cycle of HIV‐1. It cleaves immature Gag and Gag‐Pol polyproteins to create the mature protein components of an infectious HIV‐1 virion 5. This step is essential for the replication of HIV‐1. PIs were designed to target viral PR and prevent virion maturation mediated by viral PR. Mortality and morbidity associated with HIV has decreased dramatically since 1996, when PIs were introduced into cART 6. Darunavir (DRV), the most recently developed PI, is characterized by its high genetic barrier to resistance 7, 8, 9, 10; however, the emergence of DRV‐resistant HIV‐1 has now been reported 11, 12, 13.

There are two kinds of tests for the evaluation of HIV‐1 drug resistance: genotypic and phenotypic drug susceptibility tests. Genotypic drug susceptibility tests have advantages of being faster and cheaper compared with phenotypic drug susceptibility tests. However, this test estimates drug resistance indirectly and sometimes shows erroneous results if the mutation is complicated 14. In contrast, phenotypic drug susceptibility tests enable the evaluation of drug resistance directly 14, 15. Thus, phenotypic tests are more useful in combination with genotypic test 16. One disadvantage of phenotypic drug resistance tests is that they need to be conducted in a biosafety level (BSL) 3 laboratory, because in many countries, experiments using HIV‐1 are required to be conducted in BSL3 laboratories.

In this study, we established a HIV‐1 phenotypic drug susceptibility test using a lentiviral vector system that is capable of being conducted in a BSL2 laboratory, and applied the test to evaluate the antiretroviral activity of new PIs against wild‐type (WT‐lentivector) and DRV‐resistant lentiviral vectors (DRV^r^‐lentivector) as well as against chimeric lentiviral vectors containing Gag and PR of HIV‐1 CRF01_AE strains prevalent in Southeast Asia (AE‐Gag/PR‐lentivector).

## Materials and methods

### Cells

The 293T cells were maintained in Dulbecco's modified Eagle's medium (Nacalai Tesque, Kyoto, Japan) supplemented with 10% FBS.

### Lentiviral vector constructs

HIV‐1 containing four amino acid substitutions, V32I [amino acid substitution from valine (V) to isoleucine (I) at position 32]/L33F/I54M/V82I or V32I/L33F/I54M/I84V were reported as high‐level DRV‐resistant variants 17. Therefore, to generate DRV^r^‐lentivectors, four amino acid substitutions were introduced into the PR‐encoding gene of pCMV‐ΔR8.91 18, a second‐generation packaging plasmid of an lentiviral vector, by site‐directed mutagenesis using the QuikChange site‐directed mutagenesis kit (Agilent Technologies, Santa Clara, CA, USA), essentially as described previously 19. In addition, to generate AE‐Gag/PR‐lentivector, pCMV‐ΔR8.91 genes encoding HIV‐1 Gag and PR (Gag/PR genes) were replaced with the gene fragments of HIV‐1 CRF01_AE strains, as follows. The unique restriction enzyme recognition sites for *Bss*HII at the N terminus of the Gag gene and for *Cla*I at the C terminus of the PR gene were introduced into pCMV‐ΔR8.91 by site‐directed mutagenesis. CRF01_AE Gag/PR gene fragments were amplified from pNL4‐3‐derived proviral DNA containing CRF01_AE Gag/PR genes, pNL‐Luc‐E^−^‐Gag/PR45, and pNL‐Luc‐E^−^‐Gag/PR62 20, using primers containing *Bss*HII and *Cla*I recognition sites. After the enzyme digestion, CRF01_AE Gag/PR gene fragments were inserted into pCMV‐ΔR8.91 containing *Bss*HII and *Cla*I recognition sites.

### Protease inhibitors

**Figure 1:**
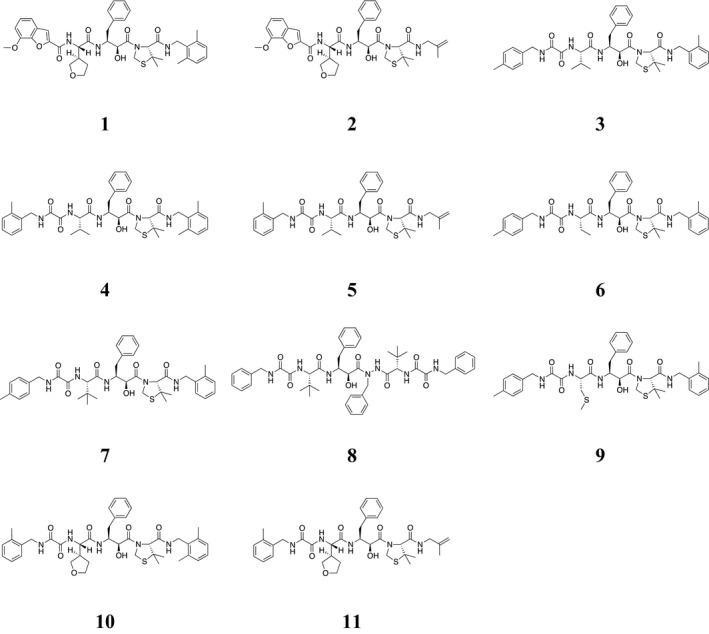
Chemical structures of 11 new protease inhibitors.

Indinavir (IDV), ritonavir (RTV), nelfinavir (NFV), amprenavir (APV), saquinavir (SQV), atazanavir (ATV), lopinavir (LPV), tipranavir (TPV), and DRV were obtained through the NIH AIDS Research and Reference Reagent Program, Division of AIDS, NIAID, NIH. In addition, new PIs **1**–**11** were synthesized as described in a previous report on allophenylnorstatine‐containing peptidomimetics 21. Briefly, the starting amines including 2‐methylbenzylamine, 2,6‐dimethylbenzylamine, or β‐methallylamine were coupled with Boc‐(*R*)‐5,5‐dimethylthiazolidine‐4‐carboxylic acid using benzotriazol‐1‐yloxy‐tris(dimethylamino)phosphonium hexafluorophosphate (BOP) or *N*‐ethyl‐*N*′‐[3‐(dimethylamino)propyl]carbodiimide hydrochloride. Removal of the protecting group using HCl/dioxane and coupling with Boc‐forms of (2*S*,3*S*)‐3‐amino‐2‐hydroxy‐4‐phenylbutylic acid (allophenylnorstatine), valine, (2*S*,3′*R*)‐tetrahydrofuranylglycine, (2*S*)‐2‐aminobutyric acid, *tert*‐leucine, *S*‐methylcysteine, or carboxylic acids including 7‐methoxybenzofuran‐2‐carboxylic acid, 4‐(methylbenzylcarbamyl)formic acid, and 2‐(methylbenzylcarbamyl)formic acid were repeated to give the crude products. Compound **8** was prepared in a similar manner to pseudosymmetric inhibitors 22. All of the products were purified by preparative HPLC with > 95% purity (Table S1) and identified by time‐of‐flight mass spectrometry (TOF‐MS) (Table S2). In addition, HIV PR inhibitory activity of the test compounds was determined based on the inhibition of a substrate (DABCYL‐Ser‐Gln‐Asn‐Tyr‐Pro‐Ile‐Val‐Gln‐EDANS) cleavage using recombinant HIV‐1 PR (Table 1). The chemical structures of 11 new PIs are shown in Fig. 1.

**Table 1:** Inhibitory activity of PIs against a synthetic substrate

Compound	% Inhibition at 5 nm (SD)	*K*_i_ (pm)
**1**	> 99	107 (± 45)
**2**	> 99	–
**3**	> 99	–
**4**	> 99	–
**5**	> 99	–
**6**	> 99	–
**7**	98.1 (± 1.9)	–
**8**	91.7 (± 2.0)	–
**9**	> 99	–
**10**	> 99	110 (± 14)
**11**	97.2 (± 1.2)	–
DRV	> 99	306 (± 29)

HIV protease inhibitory activity of the test compounds was determined based on the inhibition of a substrate (DABCYL‐Ser‐Gln‐Asn‐Tyr‐Pro‐Ile‐Val‐Gln‐EDANS) cleavage using recombinant HIV‐1 protease (a kind gift from Dr. M. Adachi). In the inhibition assay, 65 μL of 2‐(*N*‐morpholino)ethanesulfonic acid (MES)–NaOH buffer (pH 5.5) was mixed with 5 μL of the inhibitor (100 nm) dissolved in dimethylsulfoxide and 20 μL of HIV‐1 protease (200 ng·mL^−1^) in AcOH buffer (pH 5.0). The reaction was initiated by addition of 10 μL of a substrate solution. Production of cleaved EDANS substrate was monitored (excitation 335 nm/emission 500 nm) for 15 min at 37 °C, then, the kinetic slope of was calculated with or without the inhibitor. Average of the percent inhibition was obtained from more than three experiments. *K*_i_ value was determined by testing under several inhibitor concentrations fitting the velocity to Morrison's equation of tight binding inhibitor.

### Drug susceptibility test

To generate the luciferase reporter lentiviral vector, 293T cells (4 × 10^6^ cells in a 100 mm dish) were transfected with pCMV‐ΔR8.91 or the derivative, a lentiviral vector plasmid, pLenti CMV Puro LUC (w168‐1) (Addgene, Cambridge, MA, USA) 23 and a vesicular stomatitis virus G protein expression plasmid, pHit/G 24 using FuGENE HD Transfection Regent (Promega, Madison, WI, USA). Seventeen hours after transfection, 293T cells were transferred into 96‐well plate and serially diluted PIs were added in each well. Thirty‐one hours later, 293T cells, seeded as target cells 24 h prior to the infection in 96‐well plate, were infected with lentiviral vector in the culture supernatant of plasmid‐transfected cells. Twenty‐four hours after infection, luciferase activity in infected cells was measured using a Steady‐Glo Luciferase Assay System (Promega) with an LB962 microplate luminometer (Berthold, Bad Wildbad, Germany). Drug susceptibility was evaluated as a reduction in luciferase activity in infected cells. The 50% inhibitory concentration (IC_50_) of PIs for suppressing the transduction of luciferase gene was calculated from the dose–response curve using a standard function of graphpad prism 5 software (GraphPad Software, San Diego, CA, USA).

### Statistical analysis

Statistical analysis was carried out using spss Statistics Ver. 23 (IBM, Armonk, NY, USA) with Dunnett's *t* test within a one‐way ANOVA experimental design or with an unpaired *t* test.

## Results

### Establishment of a phenotypic drug susceptibility test using a lentiviral vector system

A lentiviral vector containing a luciferase reporter gene was generated from a molecular cloned, WT HIV‐1, pNL4‐3 25; therefore, it could be applied to monitor the efficiency of the single‐round replication cycle of WT HIV‐1. Namely, the production of lentiviral vector from plasmid‐transfected 293T cells could be used for monitoring the late stage of HIV‐1 replication, including the viral life‐cycle steps of RNA transcription, protein translation, viral assembly, and the release of the progeny virus. In addition, the transduction ability of the luciferase gene by the infection of a lentiviral vector to target cells could be used for monitoring the early stage of HIV‐1 replication, including the viral life‐cycle steps of viral entry into cells, encapsidation, reverse transcription, integration, RNA transcription, and protein translation. To evaluate the susceptibility of lentiviral vectors to PIs, nine clinically approved PIs were tested for their inhibitory effect on the transduction of the luciferase gene by WT‐lentivector. The results showed that IC_50_ values of seven PIs, SQV, RTV, IDV, APV, ATV, TPV, and DRV, for reducing the transduction ability of WT‐lentivector, were similar to those for inhibiting the single‐round replication of WT, luciferase reporter HIV‐1 in our previous report (Table 2) 20. Two PIs, NFV and LPV, showed relatively higher and lower IC_50_ values against WT‐lentivector than against WT HIV‐1, respectively, suggesting that the susceptibility of WT‐lentivector to these PIs was somewhat different from WT HIV‐1 (Table 2). Nevertheless, these results suggested that this phenotypic drug resistance assay was useful for evaluating the antiviral activity of PIs, although with some caution for potential differences in the susceptibility to PIs between lentiviral vector and HIV‐1.

**Table 2:** Antiretroviral activity of nine clinically approved PIs against WT‐lentivector and WT HIV‐1

Compound	IC_50_ (nm) for WT‐lentivector	IC_50_ (nm) for WT HIV‐1
SQV	1.87 ± 0.43	1.24 ± 0.11
RTV	10.39 ± 3.51	9.80 ± 0.70
IDV	7.17 ± 1.73	6.35 ± 0.64
NFV	17.52 ± 4.8*	2.91 ± 0.28
APV	7.91 ± 0.30	9.33 ± 0.82
LPV	1.60 ± 0.21*	3.12 ± 0.17
ATV	0.80 ± 0.16	0.59 ± 0.02
TPV	82.84 ± 8.69	74.69 ± 3.17
DRV	0.82 ± 0.21	0.53 ± 0.07

Antiretroviral activity of the PIs against WT‐lentivector was examined. All data are presented as mean ± standard error of six independent experiments. The 50% IC_50_ values of nine PIs for reducing the transduction ability of WT‐lentivector were compared with those for inhibiting the replication of WT HIV‐1 reported previously 20. Unpaired *t* test was used, and the IC_50_ value against WT‐lentivector was considered significantly different from that against WT HIV‐1 for *P* values < 0.05 (*).

### Evaluation of the antiretroviral activity of new PIs against WT‐lentivector

We next investigated the inhibitory effects of 11 newly designed PIs against the transduction of the luciferase gene by WT‐lentivector. Among 11 PIs tested, nine new PIs showed strong antiretroviral activity in reducing the transduction ability of WT‐lentivector (Table 3). In particular, five PIs, **1**,** 2**,** 4**,** 5,** and **10**, inhibited luciferase expression with IC_50_ values less than 2 nm (Table 3). The values of five new PIs were similar to that of DRV, the most recently developed PI, in reducing the transduction ability of WT‐lentivector. Two PIs, **3** and **7**, also inhibited luciferase expression with low IC_50_ value (2.09 nm) (Table 3); however, 2‐methylbenzyl structure at the P2′ position of **3** and **7** was potentially associated with relatively low antiretroviral activity of PIs 21. Therefore, we concluded that five PIs, **1**,** 2**,** 4**,** 5,** and **10**, may be candidates of new antiretroviral agents.

**Table 3:** Antiretroviral activity of new PIs against WT‐lentivector

Compound	IC_50_ (nm)
**1**	1.94 ± 0.60
**2**	1.16 ± 0.25
**3**	2.09 ± 0.38
**4**	1.09 ± 0.06
**5**	0.86 ± 0.20
**6**	2.26 ± 0.54
**7**	2.09 ± 0.10
**8**	42.53 ± 3.92*
**9**	2.20 ± 0.18
**10**	1.71 ± 0.39
**11**	6.80 ± 1.33*

Antiretroviral activity of PIs against WT‐lentivector was examined. All data are presented as the mean ± standard error of six independent experiments. Dunnett's *t* test was performed, and the 50% IC_50_ value of a new PI was considered significantly different from that of DRV in reducing the transduction ability of WT‐lentivector for *P* values < 0.05 (*).

### Drug susceptibility of DRV^r^‐lentivectors to DRV

We evaluated the susceptibility of DRV^r^‐lentivectors containing V32I/L33F/I54M/V82I and V32I/L33F/I54M/I84V mutations to DRV. The results showed that the IC_50_ values of DRV against the DRV^r^‐lentivectors were significantly higher than that for the WT‐lentivector (Table 4). The IC_50_ values of DRV against the V32I/L33F/I54M/V82I and V32I/L33F/I54M/I84V mutants were 12.66 ± 0.88 and 112.08 ± 27.72 nm with resistance of 15‐ and 133‐fold, respectively. These results suggested that the susceptibility of these two DRV^r^‐lentivectors to DRV were decreased as expected by the introduction of four amino acid substitutions.

**Table 4:** Antiretroviral activity of PIs against DRV^r^‐lentivectors

Compound	IC_50_ (nm)	
	V32I/L33F/I54M/V82I	V32I/L33F/I54M/I84V
DRV	12.66 ± 0.88	112.08 ± 27.72
**1**	0.18 ± 0.02*	1.30 ± 0.23*
**2**	0.08 ± 0.00*	2.43 ± 0.22*
**4**	2.15 ± 0.33*	45.40 ± 15.30*
**5**	4.85 ± 0.74*	≥ 85
**10**	0.30 ± 0.07*	1.21 ± 0.37*

Antiretroviral activity of PIs against DRV^r^‐lentivectors containing V32I/L33F/I54M/V82I and V32I/L33F/I54M/I84V mutations was examined. All data are presented as the mean ± standard error of more than six independent experiments. Dunnett's *t* test was performed, and the 50% IC_50_ value of a new PI was considered significantly different from that of DRV at reducing the transduction ability of a DRV^r^‐lentivector for *P* values < 0.05 (*).

### Antiretroviral activities of new PIs against DRV^r^‐lentivectors

We next investigated the antiretroviral activity of five new PIs, **1**,** 2**,** 4**,** 5,** and **10**, against DRV^r^‐lentivectors. Compounds **1**,** 2,** and **10**, showed significantly lower IC_50_ values in reducing the transduction ability of DRV^r^‐lentivectors (Table 4). In addition, DRV^r^‐lentivectors containing the V32I/L33F/I54M/V82I and V32I/L33F/I54M/I84V mutations were more susceptible to **1** and **10** than WT‐lentivector. In contrast, **4** and **5** showed higher IC_50_ values against DRV^r^‐lentivectors than **1**,** 2,** and **10**. In particular, the IC_50_ values of **4** and **5** against the V32I/L33F/I54M/I84V mutant were significantly higher. These results suggested that compounds **1**,** 2,** and **10** could bind to DRV‐resistant HIV‐1 PR; however, **4** and **5** were not able to maintain potent binding to highly DRV‐resistant HIV‐1 PR.

### Antiretroviral activity of new PIs against AE‐Gag/PR‐lentivector

We next subjected AE‐Gag/PR‐lentivectors to the drug susceptibility test. Five clinically approved PIs, DRV, APV, LPV, ATV, and TPV, inhibited the transduction of the luciferase gene by AE‐Gag/PR‐lentivectors effectively. Some of the IC_50_ values were relatively different from those of the five PIs for inhibiting the replication of AE‐Gag/PR‐recombinant HIV‐1 in our previous report (Table 5) 20; however, the difference might not be significant for evaluating PIs. We next conducted drug susceptibility tests for two new PIs, **1** and **10**, against AE‐Gag/PR‐lentivectors. Compounds **1** and **10** inhibited transduction of the luciferase gene by AE‐Gag/PR‐lentivectors more effectively than other PIs tested (Table 5), suggesting that **1** and **10** inhibited the replication of CRF01_AE viruses more effectively than the currently approved PIs.

**Table 5:** Antiretroviral activity of PIs against AE‐Gag/PR‐lentivectors

Compound	IC_50_ (nm)			
	AE‐Gag/PR45‐lentivector	AE‐Gag/PR45‐recombinant HIV‐1	AE‐Gag/PR62‐lentivector	AE‐Gag/PR62‐recombinant HIV‐1
DRV	5.93 ± 1.07	2.05 ± 0.26	3.88 ± 0.98	1.62 ± 0.54
LPV	33.39 ± 3.74	23.78 ± 1.17	26.96 ± 2.96	33.80 ± 5.18
ATV	9.84 ± 1.09	4.67 ± 0.37	10.41 ± 4.12	10.82 ± 1.46
TPV	359.15 ± 65.65	338.13 ± 33.02	532.70 ± 110.60	905.35 ± 214.41
**1**	4.43 ± 0.51	Not tested	3.33 ± 1.28	Not tested
**10**	4.61 ± 0.77	Not tested	3.89 ± 0.24	Not tested

Antiretroviral activity of PIs against AE‐Gag/PR‐lentivectors containing AE‐Gag/PR45 and AE‐Gag/PR62 was examined. All data are presented as the mean ± standard error of six independent experiments. The 50% IC_50_ values of four PIs in inhibiting the replication of recombinant HIV‐1 containing AE‐Gag/PR45 and AE‐Gag/PR62 were report previously 20 and shown for the comparison.

## Discussion

Phenotypic drug susceptibility testing using HIV‐1 must be conducted in a BSL3 laboratory. Although DRV has a high genetic barrier to resistance, the emergence of DRV‐resistant viruses has been reported and will presumably become a major problem in the future. In this study, we established a phenotypic drug susceptibility test that is capable of being conducted in a BSL2 laboratory by using a lentiviral vector system. In addition, the test was applied to evaluate the antiretroviral activity of new PIs against WT and DRV‐resistant HIV‐1.

Among the 11 new PIs tested, **1**,** 2**,** 4**,** 5,** and **10** showed high antiretroviral activities against WT‐lentivector (Table 3). New PIs tested in this study contain allophenylnorstatine with hydroxymethylcarbonyl isostere. The carbonyl group and the hydroxyl proton of allophenylnorstatine forms a hydrogen bond with the proton on the aspartic acid residue at amino acid position 25 and the deprotonated aspartic acid residue at the position 125, the catalytic residues of HIV‐1 PR 26. In addition, **1**,** 2,** and **10** contain tetrahydrofuranylglycine at the P2 position 27, whereas **4** and **5** contain oxamide at the P3 position, which has two carbonyl oxygen atoms as hydrogen bonding acceptors. The tetrahydrofuranyl oxygen makes a hydrogen bond with an aspartic acid residue at amino acid position 30 (D30) of HIV‐1 PR 27. Compounds **1** and **2** contain benzofurancarbonyl‐tetrahydrofuranylglycine in the P3‐P2 units, whereas **4** and **5** contain oxalyl‐valine in these units. By comparing the antiretroviral activity of the two groups, benzofurancarbonyl‐tetrahydrofuranylglycine in the P3‐P2 units are suggested to confer stronger antiretroviral activity. This might be because benzofurancarbonyl‐tetrahydrofuranylglycine in the P3–P2 units improves the balance of hydrophilic and hydrophobic moieties in the PI molecule, which presumably reflects the better membrane permeability. In addition, **10** was designed to replace valine at the P2 position of **4** with tetrahydrofuranylglycine; therefore, it contained oxalyl‐tetrahydrofuranylglycine in the P3–P2 units. Because **10** showed a strong antiretroviral activity, oxalyl‐tetrahydrofuranylglycine in the P3–P2 units may be acceptable for maintaining the antiretroviral activity of the PI.

We generated DRV^r^‐lentivectors by the introduction of amino acid substitutions. In a previous report, molecular cloned HIV‐1 mutants HIV‐1_NL4‐3_^V32I/L33F/I54M/V82I^ and HIV‐1_NL4‐3_^V32I/L33F/I54M/I84V^ showed DRV resistance of 11‐fold and 205‐fold, respectively, relative to the parental NL4‐3 17. DRV^r^‐lentivectors containing the V32I/L33F/I54M/V82I and V32I/L33F/I54M/I84V mutations showed DRV resistance of 15‐ and 133‐fold, respectively, suggesting that we could generate DRV^r^‐lentivectors that showed high levels of DRV resistance. Compounds **1**,** 2**,** 4**,** 5,** and **10** showed antiretroviral activity against the V32I/L33F/I54M/V82I mutant; however, only **1**,** 2,** and **10**, but not **4** and **5**, showed activity against the V32I/L33F/I54M/I84V mutant (Table 4). The IC_50_ values of **4** and **10** against the V32I/L33F/I54M/I84V mutant were 45.4 ± 15.30 and 1.21 ± 0.37 nm, respectively, indicating that antiretroviral activity of **10** was 37.5‐fold higher over that of **4**. It is conceivable that replacement of the valine residue (present in **4** and **5**) with tetrahydrofuranylglycine (present in **1**,** 2,** and **10**) at the P2 position of the PIs contributed to the stronger antiretroviral activity against the V32I/L33F/I54M/I84V mutant. The side chain of valine at the P2 position of **4** and **5** interacts with the S2 pocket of HIV‐1 PR by a hydrophobic interaction only; therefore, the affinity of PIs to the structurally altered S2 pocket of DRV‐resistant PR might not be maintained relative to that of WT PR. In contrast, an oxygen atom in P2 tetrahydrofuranylglycine of **1**,** 2,** and **10** formed a hydrogen bond with the amide NH of D30 on the backbone of HIV‐1 PR in addition to the hydrophobic interaction with the S2 pocket; therefore, it might be able to interact stably with the structurally altered S2 pocket of DRV‐resistant PR. The V32I/L33F/I54M/V82I mutant was rather more susceptible to **2** and **10** than WT‐lentivector. We consider that the structure of the five‐membered ring of tetrahydrofuran might fit better to the structurally altered S2 pocket of DRV‐resistant PR than to WT PR; therefore, the V32I/L33F/I54M/V82I mutant was more susceptible to **2** and **10** than WT‐lentivector.

New PIs tested in this study were designed to contain oxalyl at the P3 position, which interacts with PIs indirectly through hydrogen bonding with a bridging water molecule. These PIs are considered to be able to interact stably with drug‐resistant PR containing a structurally altered S2 pocket. However, our results suggested that tetrahydrofuranylglycine at the P2 position, rather than oxalyl at the P3 position, is important for the strong antiretroviral activity of PIs such as **1**,** 2,** and **10** against DRV^r^‐lentivectors. Therefore, further tests are required on the importance of the tetrahydrofuranylglycine residue at the P2 position for the antiretroviral activity of new PIs.

According to a previous report 20, AE‐Gag/PR‐recombinant HIV molecular clones were significantly less susceptible to clinically approved PIs than WT virus. In particular, AE‐Gag/PR62 showed nearly or more than 10‐fold resistance to nine PIs 20. Our results demonstrated that **1** and **10** were more potent against AE‐Gag/PR‐lentivectors than clinically approved PIs. HIV‐1 is characterized by extensive genetic heterogeneity. Therefore, the development of new antiretroviral drugs that are capable of inhibiting the replication of several subtypes and circulating recombinant forms of HIV‐1 is required. Our results suggested that new PIs tested in this study could inhibit the replication of not only subtype B viruses prevalent in Western countries but also CRF01_AE viruses prevalent in Southeast Asian countries 28.

In conclusion, we show here that our HIV‐1 phenotypic drug susceptibility test, which can be conducted in a BSL2 laboratory, is able to evaluate new PIs and may be applicable for use with various HIV‐1 strains. We believe that this phenotypic drug susceptibility test will be useful in the development of novel antiretroviral drugs. In addition, new PIs, **1** (KNI‐1657), **2** (KNI‐1715), and **10** (KNI‐5003) found in the present study may have the potential to be developed for clinical use in a future study.

## Author contributions

MK conceived and supervised the study; MI and MK designed experiments; MI and DO performed experiments; KH provided new tools and reagents; MI, KH, and MK analyzed the data; MI wrote the manuscript; DO, KH, and MK made manuscript revisions.

## Supporting information


**Table S1.** HPLC analysis of synthetic compounds.
**Table S2.** Time‐of‐flight mass spectrometry (TOF‐MS) data of synthetic compounds.
