# Supplementary material for: Evaluation of novel protease inhibitors against darunavir‐resistant variants of HIV type 1
Source: FEBS Open Bio. 2016 Nov 24;7(1):88–95. doi: 10.1002/2211-5463.12160 (PMC5221448; doi:10.1002/2211-5463.12160)
Supplement: Supplementary file 1 — Table S1. HPLC analysis of synthetic compounds. Table S2. Time‐of‐flight mass spectrometry (TOF‐MS) data of synthetic compounds. [file FEB4-7-88-s001.pdf]

Table S1. High-performance liquid chromatography (HPLC) analysis of synthetic compounds

Compound	Syntem A		System B	
	t_R (min)	Purity (%)	t_R (min)	Purity (%)
**1**	—	—	24.33	>99
**2**	32.43	95.7	—	—
**3**	39.05	>99	—	—
**4**	40.41	>99	—	—
**5**	36.25	>99	—	—
**6**	38.02	>99	—	—
**7**	40.50	98.3	—	—
**8**	41.24	96.9	—	—
**9**	38.45	98.5	—	—
**10**	37.17	>99	24.67	>99
**11**	32.88	>99	18.76	>99

Analytical HPLC was performed using a C18 reversed-phase column (4.6 × 250 mm; COSMOSIL 5C_{18}-AR-II) with binary solvent systems: (A) linear gradient of CH_3CN 10–90% in 0.05% aqueous trifluoroacetic acid (TFA) in 40 min, (B) linear gradient of CH_3CN 40–100% in 0.05% aqueous TFA in 40 min at a flow rate of 1.0 mL/min, detected at 220 nm.

Table S2. Time-of-flight mass spectrometry (TOF-MS) data of synthetic compounds

Compound	$[M + H]^+$		
	Formula	Calcd.	Found
**1**	$C_{41}H_{49}N_4O_8S$	757.327	757.326
**2**	$C_{36}H_{45}N_4O_8S$	693.295	693.295
**3**	$C_{39}H_{50}N_5O_6S$	716.348	716.346
**4**	$C_{40}H_{52}N_5O_6S$	730.363	730.361
**5**	$C_{35}H_{48}N_5O_6S$	666.332	666.330
**6**	$C_{38}H_{48}N_5O_6S$	702.332	702.331
**7**	$C_{40}H_{52}N_5O_6S$	730.363	730.363
**8**	$C_{47}H_{58}N_7O_8$	848.434	848.449
**9**	$C_{38}H_{48}N_5O_6S_2$	734.304	734.304
**10**	$C_{41}H_{52}N_5O_7S$	758.358	758.352
**11**	$C_{36}H_{48}N_5O_7S$	694.327	694.327

Mass spectra with electrospray ionisation, with 50% aqueous methanol as the mobile phase, were obtained using a micrOTOF-Q II spectrometer (Bruker Co., Billerica, MA).
